# Understanding overdose risk and response in permanent supportive housing: results of focus groups with tenants, staff, and leaders

**DOI:** 10.1186/s13722-025-00616-4

**Published:** 2025-11-28

**Authors:** Marina Gaeta Gazzola, Allison Torsiglieri, Stephanie Blaufarb, Lauren Velez, Patricia Hernandez, Megan A. O’Grady, Donna Shelley, David Frank, Charles M. Cleland, Kelly M. Doran

**Affiliations:** 1 Department of Emergency Medicine, NYU School of Medicine, New York, NY USA; 2 Metro Team, Corporation for Supportive Housing, New York, NY USA; 3 Department of Public Health Sciences, University of Connecticut School of Medicine, Farmington, CT USA; 4 NYU School of Global Public Health, New York, NY USA; 5 Department of Population Health, NYU School of Medicine, New York, NY USA

**Keywords:** Overdose, Permanent supportive housing, Homelessness, Opioid use, Fentanyl

## Abstract

**Background:**

Permanent supportive housing (PSH) is an evidence-based intervention for people experiencing homelessness which integrates permanent housing with voluntary support services. PSH tenants are at high risk for overdose death, yet little research to date has examined overdose in PSH. We sought to examine overdose risk and existing responses in PSH, which can shed light on opportunities for future overdose prevention efforts.

**Methods:**

We conducted focus groups with PSH tenants, staff, and leaders in New York City and New York’s Capital Region. Focus groups were recorded and professionally transcribed. Two investigators independently completed rapid turnaround qualitative analysis, completing templated summaries of each focus group and compiling key content in an analysis matrix, which a third investigator reviewed; discrepancies were resolved by consensus.

**Results:**

From October to December 2022, we held 8 focus group sessions with PSH tenants (3 focus groups, *n* = 10 total participants), staff (3 focus groups, *n* = 13), and leaders (2 focus groups, *n* = 11) grouped by role and region. Participants were diverse in age (26–67 years), gender (18 women, 16 men), race (3 Asian, 12 Black, 11 White, 5 multiracial, 3 other), and ethnicity (5 Latinx, 29 not Latinx). Analysis revealed four main themes: (1) Overdose was a large concern in PSH and created significant trauma for tenants and staff; (2) Environmental factors in PSH contributed to overdose risk; (3) There was heterogeneity in PSH buildings’ current overdose prevention efforts and adoption of harm reduction principles; and (4) Multifactorial barriers resulted in limited tenant use of opioid agonist treatment.

**Conclusions:**

Overdose is a major concern for PSH tenants, staff, and leaders. Our findings shed new light on overdose in PSH settings, providing insight into risk factors, existing responses, and barriers and facilitators to future overdose prevention efforts. These findings can inform future overdose prevention interventions within PSH.

**Trial registration:**

ClinicalTrials.gov, NCT05786222, registered 27 March 2023.

**Supplementary Information:**

The online version contains supplementary material available at 10.1186/s13722-025-00616-4.

## Background

Permanent supportive housing (PSH) is an evidence-based solution to homelessness and is a critical part of the response to homelessness in the U.S [1–5]. PSH provides permanent housing (rent-subsidized apartments) along with voluntary supportive services such as case management, and is most often targeted to individuals experiencing chronic homelessness with co-morbidities including substance use disorders (SUD) and psychiatric illnesses [2, 4]. Research has shown that PSH provides durable housing stability for a majority of its tenants [6–8]. Despite PSH’s effectiveness at resolving homelessness for its tenants, overdose rates are high in PSH, reflecting multifactorial risk factors at individual and systemic levels [9–13]. Addressing overdose in PSH is a critical element of the public health response to the overdose crisis. For example, overdoses in PSH accounted for 10% of *all* overdose deaths in New York City (NYC) in 2023, underscoring the need for tailored, place-based approaches to reduce overdose in PSH [14].

Research on the current landscape of overdose risk within PSH is limited and much has been conducted outside the U.S [15–18]. Prior work in Canada has illustrated that PSH represents a unique risk environment warranting further investigation and interventions to mitigate the overdose risk tenants face [19]. Research has identified how the risk environment is influenced by building-level factors related to the inner context of PSH (e.g., approaches to harm reduction and referral to local opioid treatment providers) and larger structural factors composing the outer context of PSH (e.g., local legislation permitting onsite overdose prevention centers) [15, 18]. Prior work has also suggested unique aspects of PSH – such as agency policies limiting visitors, tenants using drugs alone in their rooms, and changing social networks as tenants move in and out of PSH – that may affect tenant overdose risk [15, 16, 20–23].

Studies examining current approaches to reducing overdose risk and responding to overdose within PSH in the U.S. are limited. One prior study in California examined a tenant-led effort to train tenants to respond to overdose in single-room occupancy buildings (SROs)—which bear some similarities to PSH—revealing both barriers and facilitators to implementing housing-based overdose prevention and response interventions [17]. Another recent study in California examined barriers and facilitators to the use of in-room overdose prevention technology in one PSH building [24]. Key research on possible interventions to mitigate overdose risk was conducted in Canada and Europe where studies explore overdose prevention interventions such as safe use supplies, supervised consumption sites, safer supply of opioids, and integrated access to low-barrier opioid agonist treatment can attenuate overdose risk for PSH tenants [15, 18]. However, there has not been a comprehensive examination of overdose risk, response, and prevention within PSH in the U.S. Overall, large gaps remain in our understanding of overdose risk, response, and prevention within PSH in the U.S., including in New York. We conducted focus groups with PSH tenants, frontline staff, and leaders in New York, with the aim of exploring overdose risks and responses in PSH, which can inform future overdose prevention efforts.

## Methods

### Study design

The present manuscript describes a qualitative study conducted as an initial step in the development of an overdose prevention implementation intervention tailored for PSH, which is now being tested in 20 New York PSH buildings [25, 26]. We held two rounds of focus groups with PSH tenants, staff, and leaders from October to December 2022. The first round (described in the present manuscript) sought to understand overdose risks and current responses to overdose in PSH. In examining existing overdose responses, we included both immediate responses to an overdose (e.g., emergency response) as well as other efforts to lower tenant risk of and prevent fatal and nonfatal overdose (“overdose prevention”). Within our examination of overdose prevention efforts we included questions about evidence-based interventions that reduce overdose risk, including naloxone availability and opioid agonist treatment (OAT) [27–29].

We included tenants, staff, and leaders to gain a more complete picture of the topic of interest, including identifying any notable areas of convergence or divergence in ideas. Focus group findings were used to refine a set of evidence-based overdose prevention practices and implementation support that were workshopped in a second round of focus groups, which has been previously described [25]. The study was approved by the NYU Langone Health Institutional Review Board. We report study findings following the Consolidated Criteria for Reporting Qualitative Research (COREQ) [30].

### Setting

We recruited participants from nonprofit PSH agencies in NYC and New York’s Capital Region (which encompasses Albany and surrounding areas) that operate “congregate” PSH, in which PSH tenants live together in one building (versus “scattered site” PSH where tenants live “scattered” throughout the community in private market apartments). Congregate PSH buildings may house only PSH tenants or the building might be “mixed occupancy,” in which both PSH tenants and non-PSH tenants, (who receive affordable housing but not PSH services) live in the same building Study participants lived and worked in PSH buildings with a range of approximately 20 to 100 PSH tenants per building. PSH agencies sometimes own their own buildings, but more commonly the buildings are owned and operated by separate building management companies with the PSH agencies providing services to tenants in those buildings. PSH agencies offer voluntary support services that generally include a mix of case management, housing retention services, therapeutic services, and assistance (generally via referrals rather than onsite services) with mental and physical health care. Some agencies may offer specific overdose prevention and harm reduction services, although prior work has suggested that these efforts may be rare and are not standardized [31].

In 2022, the year in which our focus groups were conducted, there were over 6,000 overdose deaths in New York, 84% of which involved opioids [32, 33]. Of opioid overdose deaths, 92% involved synthetic opioids other than methadone, most commonly fentanyl [32]. Cocaine was present as a coingestant in over 50% of opioid deaths [32].

### Research team and reflexivity

Focus groups were conducted by KMD, an experienced qualitative researcher and physician with clinical and research expertise in homelessness and addiction. There was no direct clinical relationship between the participants and the interviewer. The analytic team consisted of one physician medical resident MGG with clinical and research experience in homelessness and addiction and prior experience with qualitative data analysis, and two additional study team members with master’s degrees in public health, AT and SB, who were trained in qualitative research techniques by KMD.

### Theoretical framework and interview guide

We constructed a semi-structured interview guide to examine overdose risks and responses within PSH, with questions exploring inner context, outer context, and bridging factors using the EPIS (Exploration, Preparation, Implementation, Sustainment) implementation science framework [34]. The EPIS Framework considers the inner context (e.g., organizational leadership and priorities), outer context (e.g., availability of local treatment organizations), and bridging factors (e.g., partnership between PSH agency and local buprenorphine prescriber) as related to implementation of public health or health service practices [26]. Example questions from the semi-structured interview guide are presented in Table 1, and the full interview guide is available in the supplementary material. The interview guide was created and refined with feedback from study community partners from the Corporation for Supportive Housing and the Study Advisory Board, which included individuals with lived experience of substance use and PSH tenancy along with representatives from diverse community and governmental agencies. Interview guides were tailored for the different focus group participant types (e.g., tenants vs. staff).

**Table 1:** Sample questions from semi-structured focus group interview guide

Inner Context^a^	Outer Context	Bridging Factors (for staff/leaders only)
Can you tell me about an overdose that happened in your building(s) that you witnessed or heard about? a. *Probe*: What happened? How did people respond? b. *Probe*: After this event was there any follow up / did anything change or happen? Is naloxone (also called Narcan) available in the building(s) where you live/work? a. *Probe*: How/where would someone get it? b. *Probe*: Tell me about any training that staff or tenants receive in how to use naloxone? c. *Probe* [For staff / leaders]: How did/do you communicate about naloxone availability/training/policies to staff and tenants in your building(s)? d. *Probe* [For tenants]: How did you learn about naloxone being available in the building? What is your perception about how much staff and leaders at your building(s) know about addiction and overdose? a. *Probe*: Can you tell me more about that? b. *Probe*: What type of training do staff receive related to overdose or addiction?	What services exist in your local areas to assist tenants with addiction? a. *Probe*: Where do you usually refer people for SUD needs? [Or for tenants, where do you or others usually go to get help with your substance use? ] b. *Probe*: Can you tell me what you liked or did not like about these treatment or other programs? Can you tell me about how these programs or treatments worked for [you / your tenants] or what could make them better? c. *Probe*: For specific types of services – SUD treatment, harm reduction, buprenorphine Now let’s talk about the neighborhoods where your building(s) are located. How much of an issue is drug use in the neighborhood / area around your building(s), if any? a. *Probe*: Is there visible drug use in the area around the building(s)? b. *Probe*: How does this affect building tenants, if at all?	What role do any governmental, regulatory, or financing agencies have in influencing or driving in some way what happens in your building(s)? *a. Probe*: for which agencies and how? *b. Probe*: for potential positive vs. negative influence *c. Probe*: for how funding source influences staffing, how services are administered, what can be offered, etc. How does increasing programing related to overdose prevention fit or not fit into regulatory or other requirements for permanent supportive housing? *a. Probe*: For example, are there any policies or mandates that would either promote or hinder this sort of work?

^a^*The EPIS Framework (Exploration*,* Preparation*,* Implementation*,* Sustainment) considers the inner context (e.g.*,* organizational leadership and priorities)*,* outer context (e.g.*,* availability of local treatment organizations)*,* and bridging factors (e.g.*,* partnership between PSH agency and local buprenorphine prescriber) as it relates to implementation of evidence-based public health or health service practices* [34]

### Study sample

As noted earlier, we recruited participants from nonprofit PSH agencies in NYC and New York’s Capital Region that operate congregate PSH. We prioritized selection of PSH agencies / buildings for which there were concerns related to overdose, such as buildings where overdoses had previously occurred or that served tenants likely to be at higher risk for overdose (for example, buildings targeted for individuals with a history of substance use and/or mental illness). Study participants included PSH tenants from these buildings as well as frontline staff (e.g., counselors, case managers) and senior leaders (e.g., vice presidents, chief executive officers) from the nonprofit organizations offering PSH services within these buildings. To be eligible, participants had to be English-speaking adults not incarcerated and able to provide informed consent. For tenants, we restricted eligibility to those with a history of overdose or opioid use experience, either personally or via close experience of a family member, friend, or neighbor. We held separate focus groups for each participant type to maximize the likelihood that participants would share their thoughts freely.

### Study recruitment process

We recruited participants using a purposeful sampling approach, seeking participants who would be able to provide a depth and breadth of insight related to the study questions and working to ensure participant diversity (e.g., in size/type of PSH agency, participant demographics). We invited PSH agency leaders via email and sought their recommendations for staff who might have insight aligned with focus group aims. We then asked staff for recommendations of tenants with relevant experiences. We also asked PSH buildings to hang fliers with information about the study and a phone number for interested tenants to call the study team directly. Interested potential participants met with a study coordinator to discuss the study procedures and confirm eligibility. All participants provided informed consent. We compensated participants with a $40 Visa or Amazon gift card.

### Data collection

Focus groups were conducted by secure web conference, with an option for telephone-only participation. KMD facilitated the focus groups and additional study team members attended for notetaking and technical support purposes. The facilitator first introduced themself and the study aims and invited participants to introduce themselves. Ground rules were provided for focus groups including that the content would remain confidential, that space should be made for all participants to talk, and that participants could share as much or as little as they felt comfortable with. Focus groups were audio recorded and professionally transcribed. Transcripts were deidentified prior to analysis.

### Analysis

Data was analyzed using rapid turnaround qualitative analysis methods [35, 36]. After each focus group, one study team member completed a templated summary of the discussion, using a pre-designed template based on key study aims. A second team member reviewed each templated summary and made any additions or edits as needed. Templated summaries were then used to create an analysis matrix in which data for each focus group was condensed and organized according to key domains based on the theoretical framework (e.g., inner context, outer context, bridging factors). One investigator (AT or MGG) prepared the first draft of the matrix, which the other then reviewed for agreement, with discrepancies resolved using consensus and input from a third study team member (SB) and the study principal investigator (KMD). The matrix was then used to facilitate identification of salient, commonly recurring themes and subthemes specifically related to overdose and overdose prevention, with investigators returning to focus group transcripts to identify relevant text segments. Themes and subthemes were confirmed and refined iteratively by KMD and MGG.

## Results

From October to December 2022, we convened 8 focus groups with NYC tenants (*N* = 2 focus groups), Capital Region tenants (*N* = 1), NYC staff (*N* = 2), Capital Region staff (*N* = 1), NYC leaders (*N* = 1), and Capital Region leaders (*N* = 1). In total, 34 individuals participated in a focus group, including PSH tenants (*n* = 10), staff (*n* = 13), and leaders (*n* = 11). As detailed in Table 2, participants varied in age, gender, race, and ethnicity. Focus groups varied in size from 2 to 6 participants and ranged from 50 to 90 min in duration.

We identified four main themes from the focus groups: (1) Overdose was a large concern in PSH and created significant trauma for tenants and staff; (2) Environmental factors in PSH contributed to overdose risk; (3) There was heterogeneity in PSH buildings’ current overdose prevention efforts and adoption of harm reduction principles; and (4) Multifactorial barriers resulted in limited tenant use of opioid agonist treatment. These themes as well as subthemes and illustrative quotes are summarized in Table 3 and detailed in the sections that follow. In general, we observed concordance across focus group types (e.g., focus groups with tenants versus staff versus leaders) in the main themes, with convergence of the information and examples provided by the different types of participants. In these instances, we generally refer to “participants” to mean participants from across groups. For some themes, participants with different roles had differing perspectives about the factors or mechanisms underlying the theme. For these situations, we specify which groups described certain ideas. For example, tenants and leaders expressed concern that staff were uncomfortable using naloxone, while staff and leaders described tenants being hesitant. We also observed generally that leaders sometimes reported on interventions or policies that they believed their organization was implementing related to overdose prevention or response, whereas staff and tenant interviewees shared a different perspective of how these activities were carried out on the ground that was less idealized or revealed gaps. Other points of convergence or difference across participant types are discussed as relevant in the sections that follow.

**Table 2:** Focus group participants

Focus Group Type	*n* ^*^	Characteristics^*^
PSH tenants	10	Age, mean (SD): 58.4 (4.5) years Gender: 2 women, 8 men Ethnicity: 8 not Latinx, 2 Latinx Race: 4 Black, 3 White, 2 “more than 1 race,” 1 “other”
NYC 1	5	
NYC 2	2	
Capital Region	3	
PSH staff	13	Age, mean (SD): 44 (13.8) years Gender: 10 women, 3 men Ethnicity: 11 not Latinx, 2 Latinx Race: 1 Asian, 7 Black, 2 White, 2 “more than 1 race,” 1 “other” Role: 1 Program Manager, 1 Program Supervisor, 2 Program Directors, 2 Senior Case Managers, 2 Leasing or Residence Managers, 1 Medication Manager, 1 Building Coordinator/Advocate, 1 Assistant Program Director, 1 Socialization/Recreation Advocate, 1 Supportive Housing Shift Manager Coordinator
NYC 1	5	
NYC 2	4	
Capital Region	4	
PSH leaders	11	Age, mean (SD): 47 (11) years Gender: 6 women, 5 men Ethnicity: 10 not Latinx, 1 Latinx Race: 2 Asian, 1 Black, 6 White, 1 “more than 1 race”, 1 “other” Role: 2 Executive Directors, 3 Associate Executive Directors or Assistant Vice Presidents, 2 Department or Senior Directors, 1 Chief Program Officer, 3 Program Directors
NYC	6	
Capital Region	5	

* “n” is number of participants in each focus group. Combined characteristics are shown for each participant type to preserve participant confidentiality

**Table 3:** Summary of themes

Themes	Subthemes	Illustrative Quotes
Theme 1: Overdose was a large concern in PSH and created significant trauma for tenants and staff		*In the past three years*,* I’ve known about five people that’s overdosed. Two kinda personal*,* but three that just were tenants that live in my building. So*,* I know that this opioid problem is a big problem. – Tenant 9A*
		*It [tenant overdose] can be traumatizing and can be stressful. – Staff 9A*
		*It was really frightening to see so many people die*,* and not be able to process that. My next-door neighbor died. – Tenant 5A*
Theme 2: Environmental factors in PSH contributed to overdose risk	Subtheme 2.1: Tenants using drugs alone behind closed doors was a common factor in overdose deaths	*A lot of it [drug use] they do in their rooms and they don’t do it with other people. And that’s one thing my organization used to try to tell them: have somebody else around. But a lot of times they don’t wanna do it…. – Tenant 4A*
		*But that’s my advice to anybody who’s gonna do drugs. To always expect that fentanyl is gonna be included in the drugs you get. So*,* at least try to be with somebody so you don’t have to die*,* which is*,* of course*,* why I’m still around today. - – Tenant 4B*
	Subtheme 2.2 Easy availability of an unpredictable drug supply created overdose risks	*I’m pretty sure it’s the area because it’s so prevalent and I can walk outside*,* and I can get whatever I want in like one minute. … When it’s so prevalent and easy to find you have more cases of overdoses. – Staff 6A*
		*The slightest bit of fentanyl mixed in…which we’re seeing a lot of, not only with cocaine, crack, and meth, but a lot with pills, the pressed pills that are made that are mixed with fentanyl. And people who have never had an opioid in their system, and it doesn’t take much for them to overdose. – Leader 6A*
		*We will go months without an overdose and then have five in a week. And I think that has a lot to do with access to the suppliers that our clients have. – Leader 4A*
Theme 3: There was heterogeneity in PSH buildings’ current overdose prevention efforts and adoption of harm reduction principles	Subtheme 3.1: PSH buildings had variable efforts related to naloxone distribution, training, and utilization and other harm reduction interventions	*We train clients all the time. But I can’t actually think of a single instance where the client actually Narcan’d. They just called staff and told them to come up. – Leader 1 A*
		*They offer the Narcan*,* we have it*,* they have it on the floor*,* and every floor they put five bags in a basket every day. And people be taking them as they need them. I take it with me every day – Tenant 3A*
	Subtheme 3.2: Wellness checks and other surveillance efforts underscored tensions between tenant autonomy and safety	*In my building they have an awareness program for people that are already been noticed using heroin… people that are using it [opioids] are checked on daily almost because of 10 deaths in two and a half years.* –*Tenant 5A*
		*We’ve made some changes at our front desk of how they sign in. By doing that*,* we’re able to track a lot better when someone is coming in*,* and when someone has not left for a long period of time*,* as well. If a certain amount of time has passed by we’re just gonna head up*,* and say*,* “Hey*,* just checking on you to make sure you’re okay” … I think that’s just good practice [and] shows the [tenants] how much we care about them*,* and that we actually want what’s best for them. –Leader 5B*
		*When I was caught up in the grips*,* I did not want you knocking on my door. I did not come downstairs to any of your meetings. I did not do anything you asked me to do because I was isolated in my own world. So*,* the whole key to this is the bridge that someone mentioned*,* trying to develop a rapport with those that are sicker than others. –Tenant 6A*
	Subtheme 3.3: PSH buildings differed in their interpretation of harm reduction principles	*“In the tenant handbook it basically talks about general rules and regulations within living in supportive housing where it pertains to the site. Of course*,* illicit drug use*,* no. There is something about criminal activity. But for the most part we can’t enforce that. That would mean calling the cops on our tenants. We’re not doing that.” -Staff 7A*
		*We try to have an open dialogue*,* make sure that we’re forming a therapeutic alliance… we’re not judging you for using. If you are gonna use*,* we want you to use in a safer way. So*,* that’s why we introduced the harm reduction approach*,* once we started having a lot of deaths in the building.* –*Staff 3A*
Theme 4: Multifactorial barriers resulted in limited tenant use of opioid agonist treatment	Subtheme 4.1: PSH tenants faced practical barriers to accessing substance use treatment	*Whether it’s methadone*,* or it’s MAT [Medication for Addiction Treatment]*,* or it’s ambulatory detox…all of them are very difficult to access… the outpatient is getting less and less accessible. They can’t keep workforce. Even if somebody is engaged in outpatient treatment*,* they’re gonna get different counselors or therapists from time to time*,* and that’s discouraging*,* and that’s turning away some of our folks. We have very few of our folks who are engaged in community-based treatment. –Leader 1B*
	Subtheme 4.2: Some participants expressed skepticism about opioid agonist treatment	*That’s been the hardest part*,* is that they really will direct you to the outpatient treatments [such as OAT]*,* and you have to fail at that I think three times before they’ll consider doing anything. I can’t tell you how many times I’ve seen a person who wants to go to [inpatient] treatment. They know what they need and yet nobody is listening to them. –Leader 3B*

### Theme 1: Overdose was a large concern in PSH and created significant trauma for tenants and staff

Participants described overdose as *“truly the epidemic that we’re [PSH agency] struggling with*” [Leader 2A]. Nearly all participants were aware of an overdose death among a PSH tenant in the building where they lived or worked, and many had personally responded to an overdose in PSH. At the most extreme, participants noted that responding to overdose seemed to happen *“almost every day”* [Staff 6A], whereas for others it was rarer but still recognized as a *“big problem”* [Tenant 9A] that *“can be really overwhelming sometimes”* [Staff 2B].

Participants described how tenants, staff, and leaders experienced grief and loss following overdose deaths in their buildings: “*not only us [staff] but the tenants feel the death of their fellow community members who are dying”* [Staff 7A]. One tenant described how finding one of his neighbors after they had overdosed “*really messed me up seeing him like that”* [Tenant 7A]. Another tenant described the impact of a neighbor he was close with overdosing: “*just one day*,* he was dead in the building – in this apartment. So*,* that kinda hit me hard*” [Tenant 9A]. Participants also reflected on the trauma staff felt from finding tenants who had suffered a fatal overdose “too late:”

*It’s had an incredible vicarious traumatic effect on staff because typically*,* it’s direct care staff that are finding folks. They’re usually finding folks too late. And then they’re taking that trauma home with them. With people they’ve built relationships with*,* in some cases for years*,* all of a sudden are out of the picture.* —*Leader 2A*

Building responses to support staff and tenants after a tenant overdose seemed to vary considerably. Some participants reported that their buildings had no formal notification or remembrance process, while others reported their buildings made limited gestures such as hanging a photo of the decedent in the elevator. More rarely, participants described how their organizations sponsored memorial services or even hired professional “*bereavement teams that come immediately*” [Staff 6 A] to help tenants and staff process grief. In general, tenants and staff reported desiring more support for the grief associated with responding to tenant overdose. For example, one staff member reflected, “*because of the amount of*,* number of*,* deaths that staff has experienced*,* it’s highly stressful and traumatizing. Yes*,* an email [sent to staff after a tenant dies] is very nice*,* but*,* we’re human beings and an email is not enough to provide support”* [Staff 8A].

### Theme 2: Environmental factors in PSH contributed to overdose risk

Focus group participants described factors at different levels of the environment—including the micro-environment of the PSH building (Subtheme 2.1) as well as the macro-environment related to the drug supply (Subtheme 2.2)—that they felt impacted tenant overdose risk.

#### Subtheme 2.1: Tenants using drugs alone behind closed doors was a common factor in overdose deaths

Participants discussed how, unlike homeless shelters, which are often congregate settings in which multiple individuals may share one dorm room, PSH tenants generally have their own apartment units. Nearly every participant remarked how using drugs alone behind closed doors contributed to fatal overdose in PSH. One staff member reflected, “*this year*,* there were two overdoses that ended in death that we were not able to get to because people were using it alone in their room with the door closed”* [Staff 3B]. A tenant with personal experience of overdose underscored the potential dangers of using drugs alone:

*Most of the friends that I’ve lost*,* and myself included*,* would tend to use drugs by themselves. And that’s where the problem is*,* because you don’t have anybody to induce the Narcan. It’s too late unless somebody’s there. That’s how I was saved three times. I had people there. Thank God*,* because the majority of times I was using alone. –Tenant 3B*

Participants offered explanations for why tenants might use drugs alone, including a lack of social connections (e.g., *“one person was a loner” [Leader 2B]);* desire to not have to share one’s drug supply; or preference for privacy when using drugs (“*I just got to the point where I enjoyed getting high by myself more*…*my goal was to get high alone. And I think that’s the case with a lot of people”* [Tenant 3B]).

Additional micro-environmental (i.e., at the level of the PSH building) factors that may have contributed to drug use alone are described in Subtheme 3.4.

#### Subtheme 2.2: Easy availability of an unpredictable drug supply created overdose risks

Participants frequently discussed how drug supply contamination (e.g., with fentanyl) impacted overdose: “*it’s never-ending with this fentanyl that they’re adding to all the drugs now. That’s why you have so many ODs*” [Tenant 4B]. Participants generally viewed drug selling as common in PSH buildings and surrounding neighborhoods, which both facilitated tenant substance use and created challenges for those trying to reduce or abstain from use: “*you walk out my building… everybody and their mother selling drugs on that corner”* [Tenant 7A]. Some participants reported a belief that people selling drugs specifically targeted PSH tenants for drug sales. A few participants also described observing overdoses occurring in clusters in their buildings, which they postulated might be related to the hyper-local supply (i.e., bad batches being sold in areas local to their buildings): For example, one PSH leader noted, “*We will go months without an overdose and then have five in a week. And I think that has a lot to do with access to the suppliers that our clients have*” [Leader 4A].

### Theme 3: There was heterogeneity in PSH buildings’ current overdose prevention efforts and adoption of harm reduction principles

The most common efforts related to overdose prevention in PSH described by participants were related to naloxone distribution and training. A few other harm reduction and substance use treatment interventions were described by some participants, with heterogeneity across buildings (Subtheme 3.1) Participants also discussed various forms of surveillance aimed at preventing overdose including tenant “wellness checks” (Subtheme 3.2). In general, focus group participants described a somewhat mixed approach to harm reduction, with philosophies and practicalities sometimes conflicting (Subtheme 3.3).

#### Subtheme 3.1: PSH buildings had variable efforts related to naloxone distribution, training, and utilization and other harm reduction interventions

Most participants reported their buildings had naloxone available in some capacity, but locations where it was kept and its accessibility (particularly to tenants) varied considerably. Some buildings tried to increase naloxone access by displaying it in common areas or keeping it stocked on every floor. One leader noted that their building kept naloxone *“in the same spot in every apartment*,* so even if you were in a brand-new person’s room that you hadn’t visited before*,* you would know where the Narcan was”* [Leader 3B]. This was the only leader or staff member reporting naloxone being stocked uniformly in every tenant room, though others reported that naloxone was stocked on each floor or in multiple building locations that could be accessed by tenants. At the other extreme, some participants reported that in their buildings naloxone was kept in locked offices or areas where tenants “*can’t seem to get it”* [Tenant 5A]. Some tenant participants reported not knowing where to find naloxone in their buildings.

Naloxone training approaches also differed across buildings. Generally, participants reported that core PSH agency staff had received naloxone training. However, PSH leaders noted being limited in being able to mandate naloxone trainings for certain categories of staff—in particular security staff—who were contracted employees from another organization. Gaps were also noted in training overnight staff due to availability. Participants described these gaps as critical because it was the security and other overnight staff who were often present at the times when tenants overdosed. Regarding naloxone training for tenants, while some tenants reported that they had access to naloxone and trainings in how to use it (“*here in the building*,* they do have it [naloxone] in the lobby*,* five bags of them*,* and in every floor available for everyone and classes if you wanna learn how to administer it”* [Tenant 3A]), others reported not being offered training (“*we haven’t had anything in black and white*,* like a presentation or training to learn how to use the Narcan or any of that*” [Tenant 3B]). Staff noted difficulties in engaging tenants in naloxone training: “*unfortunately since there weren’t a lot of incentives involved*,* many people stopped coming*” [Staff 8A]. There sometimes appeared to be a gap between the intentions of PSH leaders/staff and the on-the-ground experience of tenants, evident during focus groups where some of the tenants who said they did not know where to find naloxone or had not been offered training lived in the same buildings whose staff or leaders discussed the availability of naloxone and trainings for tenants.

Even among PSH programs with robust naloxone dissemination and training, there were barriers to effective naloxone use. Participants described how individuals were uncomfortable actually using naloxone:

*Despite the fact that all of our client-facing staff have been trained… I think that there is hesitation*,* that people are unsure what to do. And often*,* don’t fully understand that giving someone Narcan who isn’t overdosing is not going to do anything*,* no harm.* —Leader 6A

A tenant participant also commented on staff reticence in using naloxone: “*My staff*,* they wouldn’t Narcan him. They told me to do it and they was on the phone with 911. So*,* I think sometimes that they’re scared to perform. I’ve had to act several different times*” [Tenant 4A]. Conversely, some staff reported that tenants were uncomfortable using naloxone, although as evident in the previous quotation some tenant focus group participants themselves reported using naloxone to reverse overdoses.

While naloxone distribution and training was the most commonly discussed harm reduction intervention, participants from a few buildings reported adopting other harm reduction interventions like facilitating availability of safe use supplied for tenants. A few buildings provided tenants with fentanyl test strips. For example, one leader noted, “*I do get the sense that the ones who are engaging around it [fentanyl test strips] are using it after we talk about it*” [Leader 3A] and described how one tenant seemed to have a reduction in overdoses after they introduced the strips to him. Participants from one building described starting a harm reduction committee to better engage tenants in overdose response, and from another building reported hiring peers and substance use counselors to provide onsite services.

#### Subtheme 3.2: Wellness checks and other surveillance efforts underscored tensions between tenant autonomy and safety

When asked about overdose prevention measures, many participants highlighted the use of various types of surveillance methods, including cameras, sign-in logs, security checks, and tenant “wellness checks.” Nearly all PSH staff and leaders reported employing a version of “wellness checks” as an attempt to respond to or reduce overdoses in their buildings. As described by one staff participant, “*If we haven’t seen a tenant in about a week or if we get word from another tenant that that tenant hadn’t been seen for about a week*,* then we will key in. The basis of it would be the wellness check to make sure that the tenant is well*” [Staff 4A]. Some programs had regular intervals scheduled into their staff’s roles (e.g., daily or weekly room checks), whereas in other buildings wellness checks were ad hoc based on staff’s individual level of concern around a tenant’s substance use. Most often, these wellness checks were staff/leadership-driven, although one staff participant reported that they sometimes did wellness checks based on tenants sharing concerns about other tenants. Participants commented on the timing of wellness checks, noting that given the relatively low frequency with which they occurred they seemed just as likely to result in finding a tenant who had died from an overdose as actually preventing a fatal overdose from occurring. However, some staff and leaders also noted that “wellness checks” served purposes beyond overdose prevention such as identifying tenants who needed help with their physical or mental health. One staff participant uniquely shared a more proactive—and likely more effective—form of overdose prevention wellness check, noting that there was a tenant who himself would check in with her before using drugs, with the intention that: “*if I don’t see him within like a 20-minute span*,* I either go find him*,* or he comes to find me*” [Staff 1B]. Concerns about accessibility of drugs and tenant safety more generally additionally prompted agencies to implement more tenant and visitor surveillance, such as through installing cameras or keeping security logs of tenants and visitors. Focus group participants generally shared a belief or hope that heightened security might discourage tenants or visitors who might be engaging in drug selling or other illegal activities.

While participants of all types seemed to share the belief that wellness checks were important, they also raised concerns about how these measures might impact tenant privacy. Tenant focus group participants specifically noted that wellness checks could infringe on tenants’ independence. Staff and leaders also described how they had to balance considerations of tenant autonomy with service provision in PSH: “*while it is supportive housing*,* they [tenants] have to be majority independent. They have free will to do whatever. This is their home. We just provide support”* [Staff 7A]. As echoed by a tenant focus group participant:

*I guess what I’m really trying to say is that even if you have a drug problem*,* you have privacy. Everybody deserves privacy and respect and to be treated a certain way. And I think it can become a problem when staff think that they have the right to invade your space or kind of wanna take control of your life and so forth because we’re adults*,* right? … And so*,* it’s a hard bridge to gap between what you do with an adult who may or may not be using [drugs] and getting them help and giving them respect and space.* —*Tenant 1A*

In general, focus group participants highlighted the need to balance tenant privacy and autonomy—which are considered part of the model of PSH in which tenants have their own apartments—with service provision and tenant safety.

#### Subtheme 3.3: PSH buildings differed in their interpretation of harm reduction principles

Participants discussed various efforts their buildings or agencies used to promote a harm reduction-focused environment—including hiring people with lived experience of addiction or homelessness, hosting staff harm reduction trainings, and partnering with local harm reduction organizations.

While PSH staff who participated in the focus groups generally reported embracing a harm reduction philosophy (*“we have to meet them where they’re at”* [Staff 4A]), participants noted there was variation on the ground regarding individual staff embracing and implementing harm reduction (“*It’s all over the map in terms of staff attuned to harm reduction or their belief in it”* [Leader 1A]). Generally, leaders reported that they believed in harm reduction as a guiding philosophy, but that their staff varied more in the level to which they embraced harm reduction, both in theory and practice:

*Despite constant efforts at addressing harm reduction and trainings and attending building-based staff meetings*,* when it comes down to the services provided*,* often staff will feel that the root of all their [tenants’] problems is substance use and if they don’t get off drugs they’re never going to get their lives together. Which is the antithesis of harm reduction.* —Leader 6A

One conflict within PSH involved discrepancies between the philosophy of not requiring abstinence from substances and written rules (e.g., in tenant leases) explicitly prohibiting drug use or possession onsite. Such written rules prohibiting onsite drug use seemed to be prevalent even for buildings that otherwise endorsed a harm reduction model, sometimes due to the building management itself being operated by a different entity than the PSH service provider and/or perceived governmental regulations. One staff member explained:

If we do go into someone’s apartment for a unit inspection and there’s paraphernalia there, they’ll get a notice of violation for the lease – that you brought something illegal in here, this is illegal. In addition, we’ll try and help them, but you violated your lease. I’m sorry, but reality is you’re putting your housing in danger. – Staff 1A

Despite the written rules, many participants described an “unspoken policy” that lease language banning drug use was not enforced (“*we’re certainly not taking any eviction-related action around drug use unless it’s elevated to a level of meth production or something like that”* [Leader 2A]). Some staff and leaders noted that the “line” that would cross them into taking action against a tenant (such as eviction) would be for illegal activities that seriously and negatively impacted other tenants’ experiences in PSH, such as drug selling, drug production, or violence.

Despite any unspoken policies, PSH leaders commonly expressed concern that tenants were not disclosing their drug use to avoid breaking rules that they perceived might result in loss of their housing. One leader noted, *“it [drug use] is still an illegal activity*,* an illegal drug. We will never evict somebody because of that*,* but they know that it is illegal*,* and they feel that risk”* [Leader 1B]. Staff and leaders noted that this same fear may have influenced tenants to use alone in their units to avoid detection. In some cases, interpretation and enforcement of building rules resulted in explicit prohibition of drug use in common spaces of the building, versus a “turning a blind eye” approach to drug use in tenant rooms:

*Because we are a harm reduction agency*,* we really can’t say*,* “You can’t use in the confines of your room.” Now*,* what we can say is “You can’t use in stairwells*,*” “You can’t use in the community rooms*,*” “You can’t use anywhere in the public area.”* —*Staff 4A*

Participants discussed how past experiences with stigma associated with addiction and substance use might impact tenants’ propensity to share information about their substance use with staff or participate in related services:

*I think past experience plays a very big role in how certain [tenants] behave in general. Especially when it comes to substance use*,* the stigma that’s attached to it*,* or them being viewed as a junkie*,* or an addict*,* or someone that’s just a criminal for doing it also plays a big role because of the fact that if someone finds out that they’re using*,* later they’re afraid that they could get arrested… because of things that certainly have happened to them. And*,* so*,* instead of trusting that we’re gonna view it as a wellness issue*,* a lot of people feel as if we may view it as a criminal justice issue.* – Leader 5B

As evident from the description above, the overall finding from the focus groups was that tenants might be receiving mixed messages related to how strongly their buildings might be following harm reduction principles, and this in turn may have impacted tendency to use alone and not disclose drug use.

### Theme 4: Multifactorial barriers resulted in limited tenant use of opioid agonist treatment

Participants reported low tenant utilization of opioid use disorder (OUD) treatment with the opioid agonist therapies (OAT) methadone and buprenorphine (one trade name is Suboxone). Most staff shared that in their buildings *“there wasn’t a lot of people using Suboxone and methadone”* [Staff 7A]. Participants’ responses to questions surrounding OUD treatment revealed opportunities for treatment facilitation for interested PSH tenants and improved staff education.

#### Subtheme 4.1: PSH tenants faced practical barriers to accessing substance use treatment

A few participants highlighted barriers that PSH tenants faced to accessing SUD treatment including OAT, including transportation and geographic limitations, restrictive treatment program policies, and health insurance issues. For example, one staff member highlighted perceptions of strict attendance policies as a limitation to effective tenant engagement in outpatient addiction treatment: “*two of them [clinics]*,* if you miss two appointments*,* you’re discharged”* [Staff 1A]. A PSH leader reflected, “*Outpatient treatment – whether it’s medication assisted treatment or a recovery to abstinence – has become less attractive*,* and less accessible during my career. And that’s making recovery more difficult”* [Leader 1B]. Other participants discussed tenants struggling with insurance and administrative issues limiting access to SUD treatment.

Rarely, participants discussed how buildings attempted to reduce these barriers through in-house approaches to SUD treatment including OAT. These included having licensed clinicians onsite for scheduled visits to prescribe medication and employing medication managers to keep and distribute tenants’ medicines (including buprenorphine) directly to assist with adherence when needed. Each of the described interventions seemed generally used by a single or just a few buildings or agencies, presenting an overall picture of heterogeneity versus universal or widespread efforts.

### Subtheme 4.2: Some participants expressed skepticism about opioid agonist treatment

Participants noted that even when OAT seemed accessible to PSH tenants, they still seemed to have limited engagement with these treatments. One staff member noted, *“There’s a lot of outpatient programs – the doctor’s office is literally across the street*,* the hospital’s like two blocks away. This area’s actually a really good area for things. I wanna say that the tenants just don’t use them”* [Staff 2A]. On delving into this lack of OAT uptake, we found that many participants—particularly staff, but also some tenants—expressed skepticism about OAT, including doubts about its efficacy. For example, one staff member described methadone as “*a sketchy solution at best*,*”* noting that they were *“not convinced that this provides any long-lasting solutions*” [Staff 1A]. Another echoed, “*Suboxone – it’s kind of a temporary fix. It may curb their cravings to do drugs for a short amount of time*,* but once they’ve gotten accustomed to using the Suboxone*,* they just backtrack and start using whatever drugs of their choice”* [Staff 3A]. A few tenant participants also expressed skepticism: *“they go to these programs and they get this Suboxone but they’re also using street drugs…. I think that’s a dangerous combination myself”* [Tenant 4fA – who reported non-opioid substance use]. Not all participants were skeptical about OAT. Namely, some tenants with personal experience of prior overdose and OUD reported positive past experiences with OAT. For example: “*Methadone saved my life for the most part because I was really struggling with heroin*” [Tenant 3B] and “*Oh*,* it’s [buprenorphine-naloxone] a good drug… I stayed clean for about eight months the first time that I was involved*” [Tenant 4B].

Overall, focus group participants generally appeared to perceive inpatient “detoxification” (“detox”) and inpatient treatment as superior to outpatient treatments such as OAT. Many participants expressed views that entering an inpatient treatment setting was necessary for tenants to treat their OUD, often directly contrasting ambulatory/outpatient care with “treatment,” a term they reserved for inpatient settings.

## Discussion

This is one of the first studies to examine combined PSH tenant, staff, and leader perceptions of overdose risk and response within PSH. Our qualitative analysis revealed four main themes: (1) Overdose was a large concern in PSH and created significant trauma for tenants and staff; (2) Environmental factors in PSH contributed to overdose risk; (3) There was heterogeneity in PSH buildings’ current overdose prevention efforts and adoption of harm reduction principles; and (4) Multifactorial barriers resulted in limited tenant use of opioid agonist treatment. These themes reflect how PSH buildings’ unique inner contexts as well as the outer contexts in which they are situated shape the risk environment for tenant overdose. In most instances, there was convergence of perspectives among tenants, staff, and leaders, although there were a few situations where there seemed to be a discrepancy across the different roles. A few areas of discrepancy underscored the need for ensuring good communication across leaders/staff/tenants as well as consistent implementation of agency plans related to overdose prevention and response.

Participants considered overdose to be a large concern for PSH tenants. They highlighted both current approaches and opportunities for intervention. Participants’ concerns about overdose mirrored the results of two different studies which found that a third of PSH tenants surveyed in Washington State had witnessed an overdose in the three months prior and a majority of PSH agencies surveyed across New York reported recent overdoses among their tenants [31, 37]. Importantly, the fact that PSH tenants are at risk for overdose should not be misconstrued as an argument against PSH. Housing is a human right and PSH is the gold standard intervention for chronic homelessness, with research showing that it increases the amount of time that people are housed rather than homeless, including for individuals with concomitant drug or alcohol dependence or mental illness [38, 39]. Individuals who would remain homeless absent PSH, including both those who are unsheltered or living in homeless shelters, also have disproportionately high rates of fatal overdose [10, 40–44].

Our study identified environmental factors of PSH buildings and neighborhoods that may influence overdose risk. Similar to the concerns highlighted in a commentary by Fleming et al. (2024), participants in our study described using alone behind closed doors as a major risk factor for overdose death in PSH [19]. Simultaneously, participants described structural features of the inner context of PSH that might lead tenants to use alone or not disclose their substance use, such as written leases that explicitly forbid drug use. Even with “unspoken rules” around allowing tenant substance use, PSH tenants may fear that their housing will be in jeopardy if they seek staff services or supports for—and thus disclose—their drug use [37]. Other aspects of the risk environment that were identified in prior studies that contributed to using drugs alone among PSH tenants included features of social networks within PSH, relationships (or lack thereof) between staff and tenants, and staffing models [19, 24]. Related to the inner context of PSH, participants discussed how interventions aimed at reducing overdose could potentially infringe on the privacy and autonomy entitled to PSH tenants, causing tension between the voluntary nature of services in PSH and the desire to maximize tenant safety. Fear of potential stigma may have deterred tenants from revealing substance use or engaging in overdose prevention efforts [15]. Participants also highlighted outer contextual elements influencing overdose risk, such as an unpredictable and contaminated but readily available drug supply shared by PSH tenants.

We also identified inner and outer contextual factors that potentially influenced OAT access and uptake among PSH tenants. OAT is one of the interventions with the highest efficacy in reducing fatal and non-fatal overdose for people with OUD [28, 29, 45]. Expanding OAT access and reducing barriers to engagement are recognized by expert consensus as key features of the public health approach to reduce overdose [46]. Though several tenant focus group participants noted their own success with OAT, other focus group participants of all types revealed skepticism toward methadone and buprenorphine, which sometimes appeared rooted in stigma toward drug use and people who use drugs. In addition to suggesting the need for improved education related to the evidence supporting OAT for overdose reduction, focus group findings revealed access barriers to outpatient treatment, some of which may be remediable via targeted facilitation provided to PSH tenants but others which may require larger outer context structural changes. Our findings fit with existing research showing people experiencing homelessness are less likely than housed individuals to receive OAT and, even when they do enroll, have lower rates of treatment retention and completion [47, 48], although studies examining OAT access and engagement specifically among PSH tenants are needed. One recent survey among PSH tenants in Washington found that few participants reported engagement with OAT [37]. Our participants underscored access and engagement challenges associated with outpatient SUD treatment including but not limited to OAT that have been identified in past studies, such as administrative policies, transportation difficulties, and financial and insurance barriers [49–51]. The present study underscores how future efforts to reduce overdose in PSH should include lowering treatment barriers to OAT among PSH tenants.

Overall, we found that current approaches to preventing and responding to overdose in PSH varied across buildings and agencies, demonstrating both strengths and potentially actionable gaps. Our findings build upon the few prior studies that have examined overdose prevention and response in PSH. The literature has described building-level interventions within the inner context of PSH to engage tenants and staff directly in overdose prevention, risk reduction, and response, such as by training tenants in how to use naloxone and harm reduction tools like drug checking [16, 17, 52]. Focus group participants in our study highlighted concrete opportunities to improve naloxone training participation among staff and tenants, increase naloxone availability and ease of access, and standardize overdose response procedures including trauma-informed approaches to support staff and tenants after an overdose. Focus groups revealed a few current practices in PSH that do not have a strong evidence base for overdose prevention specifically, such as sporadic room “wellness checks.” Use of more targeted overdose detection technologies, which allow tenants to notify staff or others of intended substance use, may be more effective by providing an opportunity to check on tenants at times they are actually at risk of overdose [24]. Other potential approaches include provision of on-site supervised consumption sites [53], which have been used in PSH in Canada [15], and promotion of services such as overdose prevention hotlines [54, 55].

### Limitations

First, our study was limited to congregate PSH in New York. However, we expect most of our findings to be applicable to congregate PSH and similar congregate housing settings (e.g., SROs) elsewhere in the country given similar overall policy and programmatic contexts as related to overdose. We enhanced generalizability of our study by including New York’s Capital Region, which may more closely approximate relevant outer context factors such as limitations in SUD treatment availability seen elsewhere in the U.S. versus New York City. Second, social desirability bias may have influenced responses in the focus groups. We attempted to limit such bias by conducting separate focus groups for PSH tenants, staff, and leaders and by assuring participant confidentiality. Being able to triangulate findings across focus groups with different types of participants further enhances confidence in the study results; for example, we were able to observe when there was a mismatch between reports of PSH leaders and those of staff or tenants that perhaps reflected differences in aspiration versus experiences “on the ground.” Last, we did not conduct in-person focus groups, which may have limited our ability to engage tenants most affected by overdose (i.e., those with active, risky illicit opioid use). Focus group participants may not have been as familiar or comfortable with using virtual platforms, though we did offer an option for tenant focus group participants to join by telephone call-in to enhance accessibility and we generally observed that tenants did not face insurmountable technological barriers to participation.

## Conclusion

In this qualitative research study, we found that overdose was a major concern for PSH tenants, staff, and leaders and current overdose-related efforts revealed both innovation and opportunities. Our findings shed new light on overdose in PSH settings, providing insight into the occurrence, context, risk factors, existing responses, and barriers and facilitators to overdose prevention. These focus group findings have already been used by our research team to inform development of a set of evidence-based overdose prevention practices suitable for the PSH context [25]. Our findings could be used by PSH organizations, public health agencies, and local governments interested in addressing overdose in PSH in their communities. Future work is needed to systematically examine the implementation of overdose response and prevention practices in PSH settings.

## Supplementary Information

Below is the link to the electronic supplementary material.

Supplementary Material 1

## Data Availability

The complete datasets generated and analyzed during the current study are not publicly available due to privacy reasons, but a limited dataset may be available from the corresponding author on reasonable request.
