# Supplementary material for: Understanding overdose risk and response in permanent supportive housing: results of focus groups with tenants, staff, and leaders
Source: Addict Sci Clin Pract. 2025 Nov 28;20:91. doi: 10.1186/s13722-025-00616-4 (PMC12664209; doi:10.1186/s13722-025-00616-4)
Supplement: Supplementary file 1 — Supplementary Material 1 [file 13722_2025_616_MOESM1_ESM.docx]

**Supplementary Material**

**Focus Group (Round 1) Guide**

***Introductory Comments (by researcher)***

- Thank participants
- Remind them of study goals
- Remind them of audio recording and confidentiality measures
- Set ground rules (e.g., be respectful of all, we may stop people to give others a chance to speak, okay to “pass” on questions)
- Note that there are no right or wrong answers—we just want to know your thoughts and get input.

***Introductions***

- Start by going around the room and having everyone briefly describe their position/how long they have been in role (for staff/leaders) or how long they have lived in building (for tenants), and any other key background information/context.

***Inner Context***

1. How much of an issue is overdose in your building(s)? Is it a big issue? A small issue?
   1. *Probe*: How often do tenants or visitors have an overdose in your building(s)? Is it more common among tenants or their visitors?
   2. *Probe*: Where do people usually overdose in your building(s) (e.g., in their own rooms, in common areas)? Are they usually alone or with others?
   3. *Probe*: How many of the tenants in the building use drugs? What types of drugs are most commonly used by tenants in the building or do you think present the biggest concerns for overdose? Is use of opioids together with other substances common or a concern in the building?
2. Can you tell me about an overdose that happened in your building(s) that you witnessed or heard about?
   1. *Probe*: What happened? How did people respond?
   2. *Probe:* After this event was there any follow up / did anything change or happen?
3. [Staff/leaders] In general, would you say that your organization follows a housing first and a harm reduction model? Why or why not?
   1. *Probe:* How do you feel about harm reduction in general?
   2. *Probe:* What does “harm reduction” mean to you?
4. How do staff in the building respond if they see a tenant using drugs in the building or if they know a tenant is using drugs?
   1. *Probe:* What would happen if a tenant used drugs in the building (for example, does this cause problems with their lease)?
   2. *Probe:* Are there any written rules or policies about drug use in the building?
   3. *Probe:* Can you give me an example of a specific interaction staff have had with [you/tenants who are known to use drugs]?
5. Is naloxone (also called Narcan) available in the building(s) where you live/work?
   1. *Probe:* How/where would someone get it?
   2. *Probe:* Tell me about any training that staff or tenants receive in how to use naloxone?
   3. *Probe* [For staff / leaders]: How did/do you communicate about naloxone availability/training/policies to staff and tenants in your building(s)?
   4. *Probe* [For tenants]: How did you learn about naloxone being available in the building?
6. What other types of programs or initiatives are currently offered in your building(s) related to preventing overdose or helping people with addiction more generally, if any?
   1. *Probe:* Related to other harm reduction (e.g., drug testing strips), education, connections to treatment, etc.
   2. *Probe* [For staff / leaders]: How did/do you communicate about these policies and programs to staff and tenants in your building(s)?
   3. *Probe* [For tenants]: How did you learn about these programs?
   4. *Probe:* Were there any other programs or initiatives like this in the past?
7. Now, thinking specifically about people’s *transition* into supportive housing, what type of support is provided at the time of this transition, particularly as related to drug use or preventing overdose?
   1. *Probe:* How long have most tenants been living in your building?
   2. *Probe:* Where did most of tenants live before entering supportive housing? How does this impact their drug use or overdose risk, if at all?
8. What is your perception about how much staff and leaders at your building(s) know about addiction and overdose?
   1. *Probe:* Can you tell me more about that?
   2. *Probe:* What type of training do staff receive related to overdose or addiction?
9. Can you tell me about the interactions or relationships between staff and tenants in your building(s) in general?
   1. *Probe:* How often do staff meet with tenants? What are those meetings like?
   2. *Probe:* How often do staff go into tenants’ units? Is this common or uncommon? Is there a particular trigger for staff to enter tenants’ units?
10. How well do you think staff understand and respond to the needs of tenants who use drugs?
    1. *Probe:* How supportive do you feel staff are of tenants who use drugs?
    2. *Probe:* How do you perceive that staff feel about people who use drugs?
11. [For tenants] Theoretically speaking, how comfortable would you feel accessing harm reduction supplies (like clean needles and syringes) or Narcan from your case manager or other staff at your housing site?
    1. *Probe:* How often do you interact with your case manager/staff?
    2. *Probe:* Do you feel like you have a good rapport with staff?
    3. *Probe:* Are you comfortable discussing your use/addiction with staff?
12. What do you think might be some barriers for staff or leaders at your building(s) in terms of doing more to prevent overdose or help tenants with addiction?
    1. *Probe:* How well supported are staff in working with tenants who might be at risk for having an overdose?
13. What types of relationships do tenants have with *each other* in the building(s), if any?
    1. *Probe:* Do tenants provide each other with social support? How often do tenants interact with each other?
    2. *Probe:* Are there any tensions between tenants who do and do not use drugs? How supportive are other tenants of those tenants who use drugs?
    3. *Probe:* Are there any tenants who take on more of a social support / natural leader role in the building(s)?
    4. *Probe:* for positive vs. negative forms of social support
14. How supported [do you feel in your / are tenants in their] personal relationships in the building? For example, are there any rules related to tenants having visitors in their units? (for example, hours that visitors are allowed, rules around visitors signing in)
15. [For tenants only] What types of support do you most need to help you reduce your risk for overdose?
    1. *Probe:* Recognize that they are the experts and we want to learn from *them* what the most important supports or interventions are.
    2. *Probe:* Is there any support or programming or other help related to your drug use that you had in a *prior* place you lived that you don’t have in your current supportive housing? What’s *missing* in terms of support where you live currently?

***Outer Context***

1. What services exist in your local areas to assist tenants with addiction?
   1. *Probe:* Where do you usually refer people for SUD needs? [Or for tenants, where do you or others usually go to get help with your substance use?]
   2. *Probe:* Can you tell me what you liked or did not like about these treatment or other programs? Can you tell me about how these programs or treatments worked for [you / your tenants] or what could make them better?
   3. *Probe:* For specific types of services – SUD treatment, harm reduction, buprenorphine
2. Are there any gaps or barriers in the services related to substance use that are available for [you / your tenants]? For example, any services that [you / your tenants] need but are not available or are hard to access?
   1. *Probe:* How easy or hard is it to use the services that exist?
   2. *Probe:* Are there any barriers to using those services? (probe for specific barriers such as transportation, insurance)
   3. *Probe:* What other types of services or programs related to drug use would be useful to [you / your tenants] but do not currently exist or have barriers to using?
3. Now let’s talk about the neighborhoods where your building(s) are located. How much of an issue is drug use in the neighborhood / area around your building(s), if any?
   1. *Probe:* Is there visible drug use in the area around the building(s)?
   2. *Probe:* How does this affect building tenants, if at all?
4. In general, how do decisions about new policies and programs get made in your building(s)/organizations? Who generally makes these decisions?
   1. *Probe:* How much influence or say do *tenants* have in decisions or policies in the building(s), if any?
5. [Not for tenants] How does increasing programing related to overdose prevention fit or not fit into other priorities within your building(s) / organization?
   1. *Probe:* For staff:tenant ratios and staff capacity
6. [Not for tenants] How much does what *other* permanent supportive housing agencies are doing influence what your organization does?
   1. *Probe:* Would you be aware, for example, if another organization started a new program to prevent overdose among tenants?
7. [Not for tenants] Do you feel like you have access to the types of training that you / your staff would need related to drug use and overdose prevention? Why/why not?

***Bridging Factors [staff/leaders only]***

1. What type of role or influence do groups like Corporation for Supportive Housing (CSH) or Supportive Housing Network of New York (SHNNY) have in your work, if any?
   1. *Probe:* For influence / role in general and also as specific to substance use.
2. What role do any governmental, regulatory, or financing agencies have in influencing or driving in some way what happens in your building(s)?
   1. *Probe:* for which agencies and how
   2. *Probe:* for potential positive vs. negative influence
   3. *Probe:* for how funding source influences staffing, how services are administered, what can be offered, etc.
3. How does increasing programing related to overdose prevention fit or not fit into regulatory or other requirements for permanent supportive housing?
   1. *Probe:* For example, are there any policies or mandates that would either promote or hinder this sort of work?
4. What types of payment structures or incentives would facilitate work to expand overdose prevention in permanent supportive housing?
5. What are your thoughts on how easy or hard it is for changes to be made or new programs started in your building(s)?
   1. *Probe:* Tell me more about that
   2. *Probe:* How supportive are leaders of change / how supported do staff feel?
   3. *Probe:* What is the practical capacity to make changes or start new programs? (e.g., time, money, resource constraints)
   4. *Probe:* What do you need to feel supported in helping clients address their substance use needs?

***Closing***

- Does anyone have any other ideas to share before we end for today?
